# Adiposity trajectories from ages 11 to 22 years: 1993 Pelotas (Brazil) birth cohort

**DOI:** 10.1590/0102-311XEN030725

**Published:** 2026-01-09

**Authors:** Riceli Rodeghiero Oliveira, Luísa Silveira da Silva, Thaynã Ramos Flores, Ana Maria Baptista Menezes, Fernando César Wehrmeister, Helen Gonçalves, Denise Petrucci Gigante

**Affiliations:** 1 Programa de Pós-graduação em Epidemiologia, Universidade Federal de Pelotas, Pelotas, Brasil.; 2 University of Illinois, Champaign, U.S.A.

**Keywords:** Body Mass Index, Overweight, Obesity, Body Fat Distribution, Cohort Studies, Índice de Massa Corporal, Sobrepeso, Obesidade, Distribuição da Gordura Corporal, Estudos de Coortes, Índice de Masa Corporal, Sobrepeso, Obesidad, Distribución de la Grasa Corporal, Estudios de Cohortes

## Abstract

We examined adiposity trajectories (body mass index - BMI and body fat percentage - BF%) from ages 11 to 22 years and described them according to sex and maternal characteristics. Data were analyzed from the 1993 Pelotas (Brazil) birth cohort. BMI (n = 3,072) and BF% (n = 3,058) measurements were assessed at four time points (11, 15, 18, and 22 years). Trajectories were found using a group-based modeling approach by sex and described according to maternal characteristics using the Pearson’s chi-squared test. This study found three BMI trajectories: “always adequate”, “always with overweight”, and “always with obesity”. Approximately 40% of the cohort was classified as “always with overweight” or “always with obesity”. For BF%, three trajectories were observed among males, including one with a consistently low BF% over time, whereas women’s trajectories showed an increase in BF% from adolescence onward. Maternal overweight was associated with higher adiposity trajectories in both sexes. Men with higher family income and lower maternal education had a higher prevalence of high adiposity trajectories for both BMI and BF%. In women, those from lower-income backgrounds had a higher prevalence of the “always with overweight” or “always with obesity” trajectories. The adiposity trajectories of men and women belonging to the 1993 birth cohort suggest significant increases in body fat from ages 11 to 22 years, and even more pronounced upward trajectories when these youths’ mothers were overweight at the beginning of this trajectory.

## Abstract (pt)

Examinamos as trajetórias de adiposidade (índice de massa corporal - IMC - e percentual de gordura corporal - %GC) dos 11 aos 22 anos de idade de acordo com gênero e características maternas. Os dados foram provenientes da coorte de nascimentos de Pelotas, Rio Grande do Sul, Brasil, de 1993. As medidas de IMC (n = 3.072) e %GC (n = 3.058) foram avaliadas em quatro momentos (11, 15, 18 e 22 anos). Utilizou-se uma abordagem de modelagem baseada em grupo por sexo para identificar as trajetórias, que foram descritas de acordo com as características maternas usando o teste qui-quadrado de Pearson. Três trajetórias de IMC foram identificadas: “sempre adequado”, “sempre com sobrepeso” e “sempre com obesidade”. Cerca de 40% da coorte foi classificada como “sempre com sobrepeso” ou “sempre com obesidade”. Para a %GC, foram observadas três trajetórias entre os homens, incluindo uma com %GC consistentemente baixo ao longo do tempo, enquanto entre as mulheres, todas as trajetórias mostraram um aumento na %GC a partir da adolescência. O sobrepeso materno foi associado a trajetórias de adiposidade mais elevadas em ambos os sexos. Homens com maior renda familiar e menor escolaridade materna apresentaram maior prevalência de trajetórias de alta adiposidade tanto para IMC quanto para %GC. Entre as mulheres, aquelas de baixa renda apresentaram maior prevalência das trajetórias “sempre com sobrepeso” ou “sempre com obesidade”. As trajetórias de adiposidade de homens e mulheres pertencentes à coorte de nascimentos de 1993 sugerem aumentos significativos na gordura corporal dos 11 aos 22 anos de idade, e as trajetórias ascendentes são ainda mais pronunciadas quando as mães desses jovens apresentavam sobrepeso no início da trajetória.

## Abstract (es)

Examinamos las trayectorias de adiposidad (índice de masa corporal -IMC-, y porcentaje de grasa corporal -%GC) desde los 11 hasta los 22 años y las describimos según el sexo y las características maternas. Se analizaron los datos de la cohorte de nacimientos de Pelotas, Rio Grande do Sul, Brasil, de 1993. Las mediciones del IMC (n = 3.072) y el %GC (n = 3.058) se evaluaron en cuatro momentos distintos (11, 15, 18 y 22 años). Las trayectorias se identificaron mediante un enfoque de modelización basado en grupos por sexo y se describieron según las características maternas utilizando la prueba chi-cuadrado de Pearson. Se identificaron tres trayectorias de IMC: “siempre adecuado”, “siempre con sobrepeso” y “siempre con obesidad”. Aproximadamente el 40% de la cohorte se clasificó como “siempre con sobrepeso” o “siempre con obesidad”. En cuanto al %GC, se observaron tres trayectorias entre los hombres, incluida una con un %GC constantemente bajo a lo largo del tiempo; mientras que, entre las mujeres, todas las trayectorias mostraron un aumento del %GC a partir de la adolescencia. El sobrepeso materno se asoció con trayectorias de adiposidad más elevadas en ambos sexos. Los hombres con ingresos familiares más altos y menor nivel educativo materno presentaban una mayor prevalencia de trayectorias de adiposidad elevada tanto en el IMC como en el %GC. Entre las mujeres, aquellas procedentes de entornos con ingresos más bajos presentaban una mayor prevalencia de las trayectorias “siempre con sobrepeso” o “siempre con obesidad”. Las trayectorias de adiposidad de hombres y mujeres pertenecientes a la cohorte de nacimiento de 1993 sugieren aumentos significativos en la grasa corporal entre los 11 y los 22 años, y trayectorias ascendentes aún más pronunciadas cuando las madres de estos jóvenes tenían sobrepeso al comienzo de esta trayectoria.

## Background

Anthropometry has been the primary method to evaluate nutritional status in population studies due to its simplicity and low cost. Body mass index (BMI, calculated by dividing individuals’ body weight by their height squared - kg/m^2^) can estimate the impact of excess body fat on health ^1^ ^,^ ^2^. However, BMI fails to differentiate between fat mass and fat-free mass, which limits its ability to capture changes in body composition and fat distribution, particularly during adolescence, a period characterized by intense physiological changes ^3^ ^,^ ^4^. In this context, body fat percentage (BF%) emerges as an important complementary measure as it enables distinguish between body mass components. Its use contributes to a more accurate assessment of adiposity, especially during stages of rapid growth and shifts in fat distribution.

A growing number of studies in the literature have investigated adiposity trajectories from childhood to adulthood ^5^ ^,^ ^6^ ^,^ ^7^ ^,^ ^8^ ^,^ ^9^ ^,^ ^10^ ^,^ ^11^. By evaluating the trajectories of adiposity one may find the development of overweight and obesity over time, enabling us to examine potential determinants and health impacts. A systematic review with meta-analysis on BMI trajectories has shown that individuals with a persistent overweight trajectory have a higher risk of developing hypertension, type 2 diabetes, and dyslipidemia and increased morbidity and mortality from cardiovascular diseases throughout their lives ^12^. In the *Avon Longitudinal Study of Parents and Children* in England, trajectories with a higher BMI from ages 7 to 24 years occurred more often in children in the highest quintile of ultra-processed food consumption, suggesting that the higher consumption of such foods in childhood is associated with greater increases in adiposity from childhood to early adulthood ^5^. A study ^10^ of BMI trajectories from ages 1 to 20 years found that the increase in adiposity, especially between late adolescence and early adulthood, was associated with a harmful cardiovascular profile.

The increase in the proportion of individuals with overweight and obesity is considered a global public health problem ^13^. In Brazil, the most recent data show that the prevalence of excess weight in adolescents aged 15 to 17 years totaled 19.4%, being higher in girls (22.9%) than in boys (16%). The prevalence of obesity equaled 6.7%, being higher among girls (8%) than in boys (5.4%). Among adults, the prevalence of overweight totaled 60.3%, being higher in women (62.6%) than in men (57.5%). Moreover, the prevalence of obesity in the population totaled 25.9%, 29.5% in women and 21.8% in men ^14^. Data from Vigitel (*Risk and Protective Factors Surveillance System for Chronic Noncommunicable Diseases through Telephone Interview*) show a significant increase in the prevalence of excess weight (BMI ≥ 25kg/m^2^) in young adults aged from 18 to 24 years in Brazil from 2006 to 2023. In 2006, 20.6% of individuals in this age group had excess weight, a number that increased to 37.4% in 2023. Over the same period, the prevalence of obesity (BMI ≥ 30kg/m^2^) more than tripled, increasing from 4.4% to 13.3% ^15^.

The age of onset of overweight and obesity constitutes an important factor to be considered due to its short- and long-term impact on health. Data from a systematic review has shown that children and adolescents with obesity are about five times more likely to be individuals with obesity in adulthood ^14^. Moreover, children and adolescents with overweight and obesity have an increased risk of diabetes and mortality from cardiovascular disease in adulthood ^16^ ^,^ ^17^ ^,^ ^18^ ^,^ ^19^.

Thus, given the importance of monitoring the change in body composition throughout life (especially in the transition between adolescence and early adulthood), this study aims to examine the trajectories of adiposity from ages 11 to 22 years among the members of the 1993 Pelotas (Brazil) birth cohort and to describe them according to individuals’ sex and maternal characteristics.

## Methods

### 1993 Pelotas (Brazil) birth cohort

All hospital births during 1993 whose mothers lived in the urban area of Pelotas, Rio Grande do Sul, Brazil, were considered eligible for that study. From January 1 to December 31, 1993, daily visits were made to the five maternity hospitals in the municipality. A total of 5,265 births to children whose mothers lived in the urban area of Pelotas were eligible for the study, of which 5,249 agreed to participate, making up the original 1993 cohort. After the children were born, their mothers were interviewed using a standardized questionnaire with questions on socioeconomic, demographic, reproductive, behavioral, health care, and health issues. The newborns were examined for weight, length, and head circumference. Subsequently, follow-ups were performed with subsamples of participants aged 1, 3, and 6 months and at 1 and 4 years of age. In the follow-ups performed at 11, 15, 18 and 22 years of age, we tried to follow all participants in the original cohort. Information on follow-ups has been previously published ^20^ ^,^ ^21^ ^,^ ^22^.

Adiposity refers to the amount of fat in a place or organ of the body, and it is an indicator of the state of body fat. To analyze adiposity trajectories, the sample of this study included all participants who had data on BMI and BF% in the last four follow-ups of the cohort. 

### Body mass index

At the 11- and 15-year-old follow-ups, anthropometric measurements of weight and height were measured. Weight was obtained by averaging two measurements of the values in grams using a digital scale with a 100g precision (SECA; https://www.seca.com/). In the 18- and 22-year follow-ups, weight was assessed using a scale attached to a BodPod (BodPod Gold Standard. COSMED; https://www.cosmed.com/en/), with a 250kg capacity and a 0.1kg precision. Height at 11, 15, 18, and 22 years of age was evaluated in cm using a portable stadiometer (aluminum and wood) with a 2m capacity and a 0.1cm accuracy.

At ages 11, 15, and 18 years, age- and sex-specific cut-off points were used to define BMI categories ^23^. At age 22, BMI was classified as underweight (BMI < 18.5kg/m^2^), adequate weight (BMI ≥ 18.5 kg/m^2^-24.9kg/m^2^), overweight (≥ 25kg/m^2^-29.9kg/m^2^), and obesity (BMI ≥ 30kg/m^2^) ^24^.

### Body fat percentage

In adolescence follow-ups, in addition to anthropometric measurements of weight and height, triceps and subscapular skinfold thickness were evaluated using skinfold calipers (Cescorf; https://lojacescorf.com.br/), with a 0.1mm precision, by three sequential measurements with a 2mm acceptable error.

In the follow-ups conducted at ages 18 and 22 years, body fat was measured by air displacement plethysmography - BodPod, which evaluates the body volume of individuals (who must remain seated inside a glass fiber). It is calculated by subtracting the air volume moved by the body in relation to the empty chamber. The Lohman equation was applied to estimate BF% in adolescents, whereas the Siri equation was used for adults ^25^ ^,^ ^26^. BF% classification was based on sex and age according to references (Supplementary Material - Tables S1 and S2; https://cadernos.ensp.fiocruz.br/static//arquivo/supl-e00030725_8163.pdf); in which adolescents were classified according to Slaughter et al. ^27^ and Pollock & Wilmore ^28^.

### Covariates

Independent maternal variables were collected by a questionnaire or measurements taken in cohort follow-ups. From the perinatal study, the sex of the cohort member (male, female) and maternal skin color recorded by the interviewer (white, black, mixed-race/yellow/Indigenous) were obtained. Household income (classified in tertiles), maternal education (0-4, 5-8, 9-11, ≥ 12 years), maternal age (< 30, 30-39 and ≥ 40 years), and maternal overweight (BMI ≥ 25kg/m^2^) were recorded in the 11-year follow-up.

### Statistical analysis

The Pearson’s chi-squared test was used to compare the sample of this study and the original cohort and to describe individuals’ trajectories according to maternal characteristics.

The trajectories of BMI and BF% were found by latent class analysis using generalized structural equation modeling to identify latent subgroups based on continuous variables. The number of classes was defined based on the relative fit of the models, using the Akaike information criterion (AIC) ^29^ and Bayesian information criteria (BIC) ^30^. Initially, this method estimated class-specific marginal means of BMI and BF%, calculating the marginal predicted probabilities for each latent class based on these means. Given these estimated probabilities, the predictions of the posterior probability of class membership were used to evaluate individuals’ probability of falling into each class and to determine the expected class for participants based on whether the posterior probability was greater than 0.5. Having determined the expected class for each participant in each follow-up, it was possible to interpret the latent classes as trajectories.

The trajectories of BMI and BF% were described according to sex and maternal characteristics. All analyses were performed on Stata (https://www.stata.com), version 16.0.

### Ethical aspects

All cohort follow-up projects were approved by the Ethics Committee of the Federal University of Pelotas, registered by Official Letters n. 029/2003 (follow-up at 11 years), n. 158/2007 (follow-up at 15 years), n. 05/2011 (follow-up at 18 years), n. 1.250.366 (follow-up at 22 years). Cohort participants, or their parents, signed a consent form prior to participating in the study.

## Results

**Figure 1:**
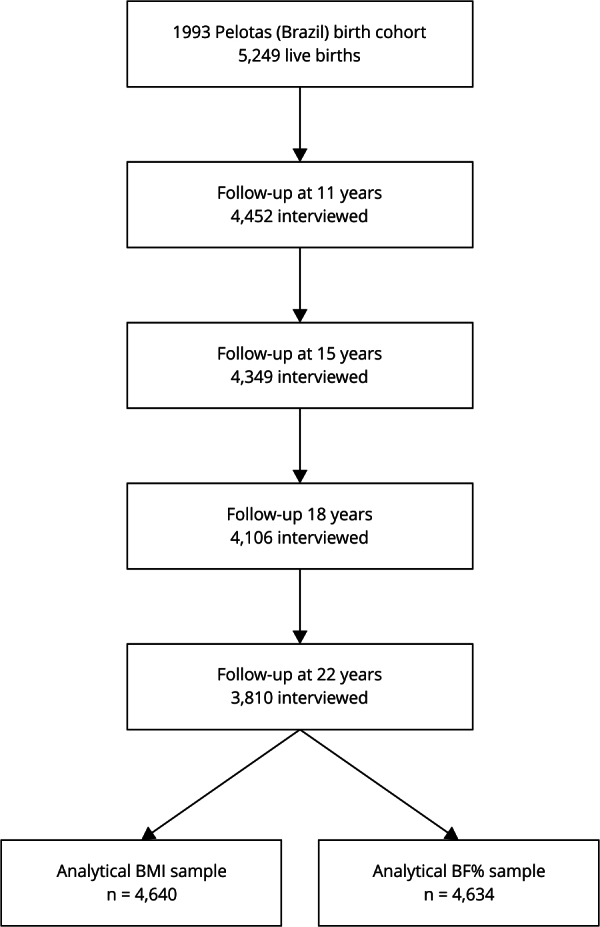
Flowchart of the study population.

Of the 5,249 participants included in the perinatal study, 87.5% were followed up at age 11 years (n = 4,452); 85.7% (n = 4,349) at age 15 years; 81.4% at 18 age (n = 4,106), and 76.3% at age 22 years (n = 3,810). Of these, 4,640 had BMI data and 4,634, BF% data, and were included in the present study (Figure 1). Regarding maternal characteristics, 77.3% of mothers were white, 53.1% were aged 30-39 years, 43.1% had 5-8 years of schooling, and 56.7% had overweight (Table 1).

BF%: body fat percentage; BMI: body mass index.

**Table 1:** Sample characteristics. 1993 Pelotas (Brazil) birth cohort (n = 4,640).

Characteristics	n (%)
Perinatal	
Maternal skin color	
White	3,585 (77.3)
Black	843 (18.2)
Mixed-race/Yellow/Indigenous	210 (4.5)
At 11 years	
Maternal age (years)	
< 30	580 (12.5)
30-39	2,464 (53.1)
≥ 40	1,595 (34.4)
Maternal education (years)	
0-4	1,144 (25.9)
5-8	1,899 (43.1)
9-11	947 (21.5)
≥ 12	421 (9.5)
Household income (tertiles)	
1 (poorest)	1,488 (33.5)
2	1,492 (33.5)
3 (richest)	1,469 (33.0)
Maternal overweight (BMI ≥ 25kg/m^2^)	
No	1,794 (43.3)
Yes	2,344 (56.7)

BMI: body mass index.

Note: missing data for maternal skin color (n = 2), maternal age (n = 1), maternal education (n = 229), household income (n = 191), and maternal overweight (n = 502).

### BMI trajectories

**Figure 2:**
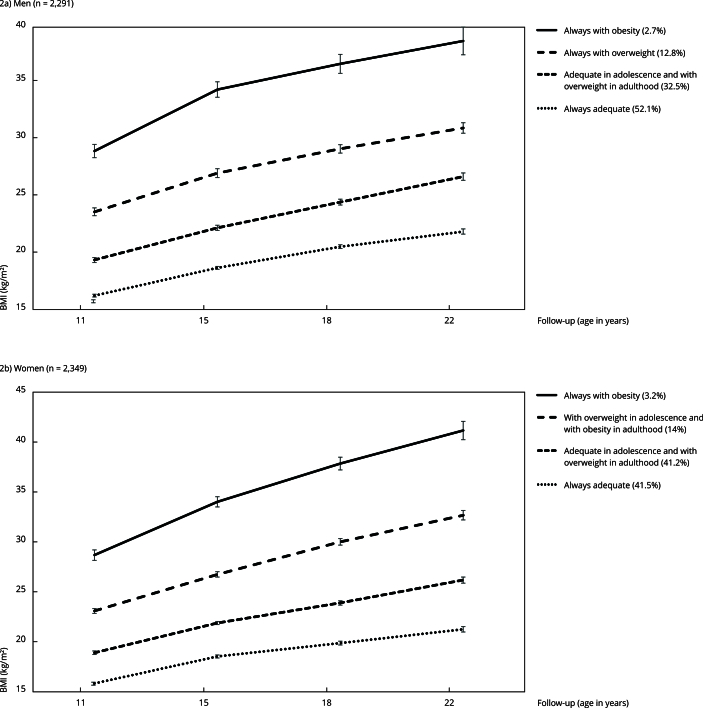
Body mass index (BMI) trajectories from 11 to 22 years. 1993 Pelotas (Brazil) birth cohort (n = 4,640).

This study found four distinct BMI trajectories from ages 11 to 22 years (Figure 2). Among men (n = 2,291), most were classified in the “always adequate” trajectory (52%), followed by “adequate in adolescence and with overweight in adulthood” (32.5%). The “always with overweight” trajectory (12.8%) included individuals with obesity at age 11 years, with overweight at 15 and 18 years, and obesity at 22 years. The “always with obesity” trajectory (2.7%) represented individuals with persistent severe obesity, showing an average BMI of 28.8kg/m^2^ (95%CI: 28.3; 29.4) at age 11 years, increasing to 38.5kg/m^2^ (95%CI: 37.3; 39.7) at age 22 years.

In women (n = 2,349), the two most prevalent trajectories referred to “always adequate” (41.5%) and “adequate in adolescence and with overweight in adulthood” (41.2%). This study classified the remaining participants as “with overweight in adolescence and with obesity in adulthood” (14.1%) and “always with obesity” (3.2%). In this last trajectory, most participants showed severe obesity up to 18 years and developed grade 3 obesity by age 22 years (average BMI: 41.2, 95%CI: 40.2; 42.1). Table 2 shows the mean BMI values by trajectory. 

**Table 2:** Body mass index (BMI) measures in each follow-up according to BMI trajectories from ages 11 to 22 years. 1993 Pelotas (Brazil) birth cohort (n = 4,640).

Follow-up	Men (n = 2,291)				Women (n = 2,349)			
	Always adequate	Adequate in adolescence and with overweight in adulthood	Always with overweight	Always with obesity	Always adequate	Adequate in adolescence and with overweight in adulthood	With overweight in adolescence and with obesity in adulthood	Always with obesity
	Mean (SD)	Mean (SD)	Mean (SD)	Mean (SD)	Mean (SD)	Mean (SD)	Mean (SD)	Mean (SD)
At 11 years	16.2 (0.06)	19.3 (0.11)	23.5 (0.17)	28.9 (0.30)	15.8 (0.08)	18.9 (0.09)	23.1 (0.13)	28.7 (0.27)
At 15 years	18.6 (0.07)	22.1 (0.12)	26.9 (0.20)	34.2 (0.34)	18.6 (0.08)	21.9 (0.08)	26.8 (0.13)	34.0 (0.26)
At 18 years	20.5 (0.08)	24.4 (0.13)	29.0 (0.19)	36.5 (0.42)	19.9 (0.10)	23.9 (0.11)	30.0 (0.17)	37.9 (0.32)
At 22 years	21.8 (0.11)	26.6 (0.16)	30.9 (0.23)	38.5 (0.62)	21.3 (0.14)	26.2 (0.16)	32.7 (0.24)	41.2 (0.47)

SD: standard deviation.

Table 3 describes sex-stratified maternal characteristics according to BMI trajectories. Regarding maternal skin color, white mothers predominated across all trajectories for men and women, with slightly higher proportions in trajectories associated with higher BMI. The proportion of black mothers was the highest in the “always adequate” trajectory (19.1% in men and 18.1% in women), decreasing in the “always with overweight” and “always with obesity” trajectories. However, these differences were not statistically significant.

**Table 3:** Maternal and cohort member characteristics according to body mass index (BMI) trajectories. 1993 Pelotas (Brazil) birth cohort (n = 4,640).

Variables	Men (n = 2,291)				Women (n = 2,349)			
	Always adequate	Adequate in adolescence and with overweight in adulthood	Always with overweight	Always with obesity	Always adequate	Adequate in adolescence and with overweight in adulthood	With overweight in adolescence and with obesity in adulthood	Always with obesity
	n (%)	n (%)	n (%)	n (%)	n (%)	n (%)	n (%)	n (%)
Perinatal								
Maternal skin color	p = 0.120				p = 0.920			
White	912 (76.5)	589 (72.2)	232 (79.5)	53 (85.5)	756 (77.7)	732 (75.6)	250 (75.7)	61 (80.2)
Black	228 (19.1)	114 (15.3)	49 (16.8)	5 (8.1)	176 (18.1)	194 (20.0)	65 (19.7)	12 (15.8)
Mixed-race/Yellow/ Indigenous	53 (4.4)	41 (5.5)	11 (3.8)	4 (6.5)	41 (4.2)	42 (4.3)	15 (4.6)	3 (4.0)
At 11 years								
Maternal age (years)	p = 0.990				p = 0.410			
< 30	154 (12.9)	94 (12.6)	36 (12.3)	8 (12.9)	117 (12.0)	130 (13.4)	32 (9.7)	9 (11.8)
30-39	630 (52.9)	401 (53.9)	153 (52.4)	33 (53.2)	513 (52.7)	508 (52.4)	179 (54.2)	47 (61.8)
≥ 40	408 (34.2)	249 (33.5)	103 (35.3)	21 (33.9)	344 (35.3)	33 (34.2)	119 (36.1)	20 (26.3)
Maternal education (years)	p < 0.001				p = 0.540			
0-4	328 (29.4)	173 (24.4)	52 (18.3)	13 (21.7)	235 (25.5)	246 (26.5)	76 (23.7)	21 (30.0)
5-8	495 (44.3)	272 (38.4)	129 (45.4)	24 (40.0)	410 (44.5)	389 (41.8)	151 (47.2)	29 (41.4)
9-11	215 (19.3)	175 (24.7)	67 (23.6)	17 (28.3)	201 (21.8)	195 (21.0)	61 (19.1)	16 (22.9)
≥ 12	79 (7.1)	89 (12.6)	36 (12.7)	6 (10.0)	76 (8.2)	99 (10.7)	32 (10.0)	4 (5.7)
Household income (tertiles)	p < 0.001				p = 0.530			
1 (poorest)	437 (38.7)	217 (30.4)	68 (23.9)	14 (22.9)	321 (34.6)	304 (32.4)	107 (33.0)	20 (28.2)
2	364 (32.2)	243 (34.0)	94 (33.1)	19 (31.2)	310 (33.4)	317 (33.8)	113 (34.9)	32 (45.1)
3 (richest)	328 (29.1)	254 (35.6)	122 (43.0)	28 (45.9)	298 (32.1)	316 (33.7)	104 (32.1)	19 (26.8)
Maternal overweight (BMI ≥ 25kg/m^2^)	p < 0.001				p < 0.001			
No	548 (52.6)	263 (39.6)	70 (26.0)	10 (18.5)	475 (54.7)	337 (38.6)	79 (26.3)	12 (18.2)
Yes	494 (47.4)	402 (60.4)	199 (74.0)	44 (81.5)	394 (45.3)	536 (61.4)	221 (73.7)	54 (81.8)
Total	1,193 (52.0)	744 (32.5)	292 (12.8)	62 (2.7)	974 (41.5)	969 (41.2)	330 (14.1)	76 (3.2)

Note: men - missing data for maternal age (n = 1), maternal education (n = 121), household income (n = 103), and maternal overweight (n = 261). Women - missing data for maternal education (n = 108), household income (n = 88), and maternal overweight (n = 241).

As for maternal education, men in the “always adequate” trajectory had the highest proportions of mothers with lower educational attainment: 29.4% had 0-4 years of schooling and 44.3% had 5-8 years, whereas only 7.1% had ≥ 12 years of education. In the “always with overweight” trajectory, 63.7% of mothers had 0-8 years of schooling and only 12.7% had ≥ 12 years. Women showed a similar pattern, although the differences between trajectories were not statistically significant.

Regarding household income, 38.7% of men in the “always adequate” trajectory were in the lowest income tertile, whereas 29.1% were in the highest. In the “always with overweight” and “always with obesity” trajectories, a higher proportion of participants were in the highest income tertile (43% and 45.9%, respectively), whereas 23.9 and 22.9% were in the lowest tertile, respectively. Among females, this pattern was not observed, and no statistically significant differences were found. Maternal overweight was more prevalent in the trajectories associated with higher BMI. In men, 74% in the “always with overweight” trajectory and 81.5% in the “always with obesity” trajectory had overweight mothers. In women, the corresponding proportions totaled 73.7 and 81.8%, respectively.

### Body fat percentage trajectories

**Figure 3:**
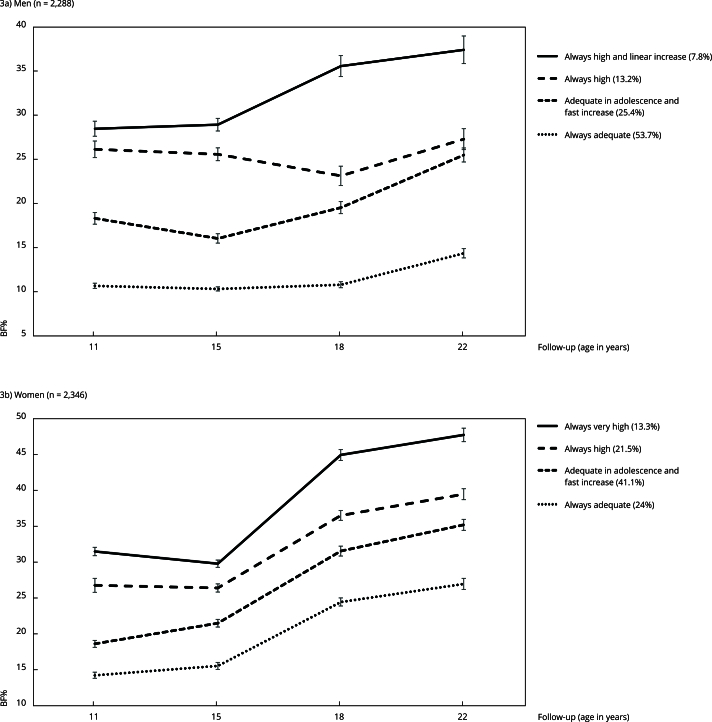
Body fat percentage (BF%) trajectories from 11 to 22 years. 1993 Pelotas (Brazil) birth cohort (n = 4,634).

Figure 3 shows four BF% trajectories for both sexes. In men (n = 2,288), the trajectories were: “always adequate”, with 53.7% of the participants classified as adequate BF% from ages 11 to 22 years; “adequate in adolescence and fast increase” (25.4%), showing a significant increase in the average BF% from age 15 years (16.0, 95%CI: 15.5; 16.6) to age 22 (25.5, 95%CI: 24.7; 26.3); “always high” (13.2%), in which participants always showed high BF%, although we could observed a small decrease in the average from ages 15 years (25.6, 95%CI: 24.9; 26.3) to 18 years (23.1, 95%CI: 22.1; 24.2); and “always high and linear increase” (7.8%), showing high BF% since age 11 years (average BF%: 28.5, 95%CI: 27.6; 29.3), linearly increasing up to age 22 years (average BF%: 37.4, 95%CI: 35.8; 39.0). In women (n = 2,346), all trajectories showed an upward trend, especially from adolescence to early adulthood. The trajectories were: “always adequate” (24%), “adequate in adolescence and fast increase” (41.1%), “always high” (21.5%), and “always very high” (13.3%), which was the trajectory that stood out for the fastest increase from ages 15 years (average BF%: 29.8, 95%CI: 29.3; 30.3) to 18 years (average BF%: 45.0, 95%CI: 44.2; 45.7) when compared to other trajectories. Table 4 describes the distribution of body fat means in each follow-up according to BF% trajectories from ages 11 to 22 years.

**Table 4:** Body fat percentage (BF%) measures in each follow-up according to BF% trajectories from 11 to 22 years. 1993 Pelotas (Brazil) birth cohort (n = 4,634).

Follow-up	Men (n = 2,288)				Women (n = 2,346)			
	Always adequate	Adequate in adolescence and fast increase	Always high	Always high and linear increase	Always adequate	Adequate in adolescence and fast increase	Always high	Always very high
	Mean (SD)	Mean (SD)	Mean (SD)	Mean (SD)	Mean (SD)	Mean (SD)	Mean (SD)	Mean (SD)
At 11 years	10.7 (0.15)	18.3 (0.34)	26.2 (0.48)	28.5 (0.44)	14.3 (0.22)	18.6 (0.24)	26.8 (0.49)	31.5 (0.30)
At 15 years	10.3 (0.12)	16.1 (0.27)	25.6 (0.37)	28.9 (0.36)	15.6 (0.25)	21.5 (0.27)	26.4 (0.29)	29.8 (0.26)
At 18 years	10.8 (0.18)	19.6 (0.35)	23.1 (0.56)	35.6 (0.61)	24.5 (0.29)	31.6 (0.35)	36.5 (0.35)	45.0 (0.39)
At 22 years	14.4 (0.27)	25.5 (0.41)	27.3 (0.60)	37.4 (0.80)	27.0 (0.39)	35.2 (0.39)	39.5 (0.39)	47.8 (0.48)

SD: standard deviation.

Table 5 shows sex-stratified maternal sociodemographic characteristics according to BF% trajectories. Maternal skin color was significantly associated with BF% trajectories in both sexes. In men, the “always adequate” trajectory included 74.5% of white mothers and 20.5% of black ones. The highest proportions of white mothers occurred in the “always high” (83.1%) and “always high with linear increase” (82.6%) trajectories. In women, although white mothers predominated across all trajectories, the “always adequate” trajectory included 70.3% of white and 24.7% of black mothers.

**Table 5:** Maternal and cohort member characteristics according to body fat percentage (BF%) trajectories. 1993 Pelotas (Brazil) birth cohort (n = 4,634).

Variables	Men (n = 2,288)				Women (n = 2,346)			
	Always adequate	Adequate in adolescence and fast increase	Always high	Always high and linear increase	Always adequate	Adequate in adolescence and fast increase	Always high	Always very high
	n (%)	n (%)	n (%)	n (%)	n (%)	n (%)	n (%)	n (%)
Perinatal								
Maternal skin color	p = 0.002				p = 0.005			
White	915 (74.5)	471 (81.1)	250 (83.1)	147 (82.6)	395 (70.3)	756 (78.4)	398 (78.8)	247 (78.9)
Black	252 (20.5)	84 (14.5)	38 (12.6)	22 (12.4)	139 (24.7)	173 (18.0)	84 (16.6)	51 (16.3)
Mixed-race/Yellow/ Indigenous	62 (5.0)	25 (4.3)	13 (4.3)	9 (5.0)	28 (5.0)	35 (3.6)	23 (4.6)	15 (4.8)
At 11 years								
Maternal age (years)	p = 0.150				p = 0.740			
< 30	170 (13.8)	72 (12.4)	34 (11.3)	16 (9.0)	70 (12.4)	130 (13.5)	57 (11.3)	31 (9.9)
30-39	652 (53.1)	316 (54.6)	146 (48.5)	101 (56.7)	297 (52.8)	509 (52.8)	269 (53.3)	171 (54.6)
≥ 40	407 (33.1)	191 (33.0)	121 (40.2)	61 (34.3)	196 (34.8)	326 (33.8)	179 (35.5)	111 (35.5)
Maternal education (years)	p < 0.001				p = 0.260			
0-4	375 (32.3)	104 (19.1)	49 (16.7)	37 (21.9)	144 (26.7)	254 (27.8)	105 (21.7)	75 (25.0)
5-8	499 (43.0)	235 (43.2)	113 (38.4)	73 (43.2)	238 (44.2)	400 (43.8)	216 (44.5)	123 (41.0)
9-11	209 (18.0)	140 (25.7)	81 (27.5)	43 (25.4)	114 (21.2)	176 (19.3)	112 (23.1)	71 (23.7)
≥ 12	77 (6.6)	66 (12.1)	51 (17.4)	16 (9.5)	43 (8.0)	84 (9.2)	52 (10.7)	31 (10.3)
Household income (tertiles)	p < 0.001				p < 0.001			
1 (poorest)	475 (40.6)	152 (27.6)	65 (22.0)	43 (25.3)	204 (37.4)	325 (35.3)	131 (26.7)	91 (30.1)
2	389 (33.2)	182 (33.0)	84 (28.5)	64 (37.6)	187 (34.3)	297 (32.2)	166 (33.9)	121 (40.1)
3 (richest)	306 (26.2)	217 (39.4)	146 (49.5)	63 (37.1)	154 (28.3)	299 (32.5)	193 (39.4)	90 (29.8)
Maternal overweight (BMI ≥ 25kg/m^2^)	p < 0.001				p < 0.001			
No	547 (50.9)	201 (38.9)	97 (34.9)	45 (28.3)	293 (58.0)	381 (44.4)	164 (35.5)	65 (23.2)
Yes	527 (49.1)	316 (61.1)	181 (65.1)	114 (71.7)	212 (42.0)	477 (55.6)	298 (64.5)	215 (76.8)
Total	1,229 (53.7)	580 (25.3)	301 (13.2)	178 (7.8)	563 (24.0)	965 (41.1)	505 (21.5)	313 (13.3)

BMI: body mass index.

Note: men - missing data for maternal age (n = 1), maternal education (n = 120), household income (n = 102), and maternal overweight (n = 260). Women - missing data for maternal education (n = 108), household income (n = 88), and maternal overweight (n = 241).

Regarding maternal education, men in the “always adequate” trajectory had the highest proportions of mothers with low schooling: 32.3% had 0-4 years and 43% had 5-8 years of education. In contrast, in the “always high” trajectory, 44.9% of mothers had nine or more years of education. Among females, a similar pattern was observed, although the differences were not statistically significant.

Regarding household income, 40.6% of men in the “always adequate” trajectory belonged to the lowest income tertile, whereas only 26.2% of them, to the highest one. In the “always high” trajectory, 22% were in the lowest and 49.5% in the highest income tertile. In women, 37.4% in the “always adequate” trajectory were in the lowest tertile and 28.3% in the highest, whereas in the “always high” trajectory, 26.7% were in the lowest and 39.4% in the highest income tertile.

Maternal overweight was also significantly associated with higher BF% trajectories. In men in the “always high” and “always high with linear increase” trajectories, 65.1 and 71.7% had mothers with overweight, respectively. In women, these proportions totaled 64.5 and 76.8%, respectively.

## Discussion

This study investigated adiposity trajectories from ages 11 to 22 years in the 1993 Pelotas (Brazil) birth cohort, finding sex-specific patterns and their associations with maternal characteristics. In both sexes, we observed BMI trajectories consistently classified as “always with overweight” or “always with obesity”. Elevated BMI trajectories were associated with maternal overweight in men and women. In men, overweight or obesity trajectories showed greater prevalence in the highest household income tertile. Regarding BF% trajectories, a marked increase occurred during adolescence and early adulthood, particularly in women. Higher adiposity trajectories were associated with higher family income, white skin color, and maternal overweight.

Evaluating adiposity trajectories across the life course enables a better understanding of the relationships between determinants and the long-term effects of excess adiposity. Obesity in childhood and adolescence is positively associated with a higher likelihood of obesity in midlife ^31^. Our study found two groups with consistently elevated BMI trajectories. Notably, in the “always with obesity” group, women fell into the grade 3 obesity category at age 22 years, which is particularly concerning.

Adolescence and early adulthood constitute critical periods for the accumulation of excess weight. Disparities related to skin color and educational level tend to increase in these stages ^32^. Trends in overweight and obesity vary according to socioeconomic and demographic factors, and these differences are particularly pronounced across countries. Analyses of demographic and health survey data from low- and middle-income countries (including Brazil) from 2010 to 2019 have shown that obesity is associated with socioeconomic characteristics such as income and education level ^13^.

Overall, overweight and obesity tend to be more prevalent in individuals with lower education levels and from lower socioeconomic strata ^33^. However, other factors may influence this relationship during childhood and adolescence. Parental socioeconomic status is directly associated with the risk of overweight and obesity in children. Systematic review evidence suggests that in high-income countries, families with higher socioeconomic status are more likely to have children with lower risk of excess weight. In contrast, in developing countries, higher socioeconomic status is associated with a greater likelihood of children with overweight or obesity ^34^ ^,^ ^35^.

In this context, this study found an association between adiposity trajectories and household income. This association occurred for both sexes in the BF% trajectories and, in the case of BMI, in men. In the high BF% trajectories, a higher prevalence occurred in individuals in the highest income tertile. In line with these findings, a study using data from a consortium of Brazilian birth cohorts from Ribeirão Preto, Pelotas, and São Luís (RPS Cohorts) found that the highest prevalence of overweight and obesity occurred in children and adolescents in the highest income tertile, whereas in adults, more commonly in those with lower income ^36^.

We also observed sex-specific differences in adiposity trajectories and their associations with maternal characteristics. In men, maternal education and household income were associated with BMI and BF% trajectories. Adolescents with better socioeconomic conditions, especially boys, were more likely to have an increase in body fat indicators ^37^.

In this study, BMI and BF% trajectories were associated with maternal overweight in both sexes. A systematic review has indicated that maternal overweight plays a key role in the development of childhood obesity, regardless of whether it occurs before conception or during children’s early life ^38^. The mechanisms underlying the intergenerational transmission of obesity are yet to be fully understood but potential contributing factors include the prenatal environment, genetic predisposition, and shared family environment. This environment encompasses lifestyle and health behaviors, such as diet and physical activity, which directly influence the risk of overweight and obesity ^39^ ^,^ ^40^ ^,^ ^41^ ^,^ ^42^.

A strength of this study lies in its use of two methods to assess nutritional status at four points across the life course. Furthermore, the transition from adolescence to adulthood is a critical period marked by age-specific changes in adiposity that may have distinct health implications. The joint interpretation of BMI and BF% more comprehensively assesses adiposity trajectories, especially considering that individuals with BMI values within the normal range may still show high BF% and stand at risk for adverse health outcomes. Therefore, by incorporating BMI and BF% as outcomes, this study enhances its ability to detect meaningful patterns in adiposity trajectories.

However, a limitation of this study concerns its use of different methods to measure BF% over time. It estimated BF% during adolescence using skinfold thickness measurements, whereas at ages 18 and 22 years, it assessed body composition by air displacement plethysmography. Although epidemiological studies widely use these validated methods, they are based on different technical principles and show varying degrees of accuracy, which may compromise the direct comparability of values across time points. Skinfold measurements tend to underestimate BF%, especially in individuals with higher adiposity ^3^, and fall subject to inter-rater variability and the limitations of predictive equations. Additionally, obtaining accurate measurements can be especially challenging in adolescents with overweight or obesity ^4^, increasing the risk of differential misclassification in participants. In this study, considering that elevated BF% occurred from early adolescence in both sexes, it is likely that any potential underestimation due to the use of skinfolds failed to substantially affect the overall findings. Nevertheless, this methodological limitation underscores the need for caution when interpreting comparisons across assessment periods. In this context, the analysis of trajectories based on BMI may be more appropriate for evaluating changes in adiposity over time.

In conclusion, this study found four adiposity trajectories based on BMI from adolescence to young adulthood, two of which (“always with overweight” and “always with obesity”) were associated with higher household income in men and with maternal overweight in both sexes. It also found four BF% trajectories, two of which always showed high or very high levels of adiposity, which were associated with maternal skin color, household income, and maternal overweight in both sexes. This study found significant increases in BMI and BF% from ages 11 to 22 years in cohort participants. Our findings contribute to the understanding of adiposity trajectories across the life course and their potential health impacts. They emphasize adolescence as a critical developmental period, essential for the adoption of healthy behaviors and the implementation of early interventions aimed at preventing excess weight and its long-term consequences.

## Data Availability

The research data are available upon request to the corresponding author.
